# Stronger Activation of the Right Primary Motor Cortex in the Early Processing Stage of Negative Emotional Scenes Stimulation

**DOI:** 10.1155/da/2386445

**Published:** 2026-04-20

**Authors:** Haijiang Meng, Xiaoxiao Zhang, Jian Zhang, Chaoying Zheng

**Affiliations:** ^1^ School of Physical Education, Shaoxing University, Shaoxing, Zhejiang, China, usx.edu.cn; ^2^ School of Psychology, Shanghai University of Sport, Shanghai, China, sus.edu.cn; ^3^ China Table Tennis College, Shanghai University of Sport, Shanghai, China, sus.edu.cn

**Keywords:** motor cortical excitability, motor evoked potential, negative emotion, primary motor cortex, transcranial magnetic stimulation

## Abstract

**Background:**

To identify threatening emotional information in a natural scene and form a rapid behavioral response, which is crucial for individual survival. The motor cortex is thought to play a key role in this process. However, little research has addressed differences in the motor system responses to valence‐specific emotional stimuli across multiple temporal windows.

**Methods:**

Twenty‐eight participants were asked to complete the emotional picture classification task, and then triggered the single‐pulse transcranial magnetic stimulation in a specific time window after various emotional pictures were presented, to evaluate the excitability of left and right primary motor cortex (M1) in different time windows after emotional picture presentation.

**Results:**

In the early time window (150 ms), the right side of M1 was more excited when viewing negative emotional pictures than neutral emotional pictures and positive emotional pictures, while in the late time window (350 ms), the right side of M1 was more excited when viewing negative emotional pictures than neutral emotional pictures, and the excitability of right‐side M1 was higher when viewing positive and negative emotion pictures, but there was no significant difference in the excitability of right‐side M1 induced by emotional pictures in the medium‐term time window (250 ms).

**Conclusion:**

The response of motor system to emotional stimulation is mainly reflected in the right hemisphere. In the early time window, negative emotional stimuli elicited a stronger response in right M1, whereas in the late time window, positive and negative emotional stimuli elicited a similar response in right M1. These findings may shed light on the neural basis of emotional motor biases, which could be disrupted in mood disorders like depression and anxiety.

## 1. Introduction

Negative emotional scene stimuli can induce faster motor responses [1, 2] and greater force output [3], both of which are critical for avoiding potential threats and ensuring survival [4]. The motor cortex is believed to be a central region involved in this process [5, 6]. Human electroencephalography studies have shown that neural activity in the primary motor cortex (M1) is rapidly modulated within the early stage of negative emotion processing (100–200 ms), consistent with the evolutionary mechanism of prioritizing threats [7, 8]. However, since electroencephalography cannot distinguish between excitatory and inhibitory processes within the motor cortex, traditional methods have not clarified the functional nature of motor activation induced by negative emotional stimuli. Transcranial magnetic stimulation (TMS), which allows for millisecond resolution quantification of motor evoked potentials (MEPs), enables the characterization of M1 responses to emotional stimuli across three temporal phases: early attention (150 ms, threat detection), intermediate integration (250 ms, emotional evaluation), and late regulation (350 ms, behavioral selection) [9, 10]. Several studies have reported significantly enhanced activation of the left M1 approximately 300 ms after the onset of both negative and positive emotional scenes [5, 11]. Only one study has found that the activation of the left M1 is stronger at 150 ms following the presentation of negative emotional scene stimuli [12]. Nonetheless, whether the right M1 exhibits stronger activation during the early processing stage of negative emotional scenes remains unclear.

Previous human studies have demonstrated that emotional perception and expression are functionally lateralized across the cerebral hemispheres [13, 14]. Two primary hypotheses have been proposed to explain this asymmetry: the right hemisphere hypothesis and the valence hypothesis. The right hemisphere hypothesis suggests that the right hemisphere has a dominant role in emotional processing relative to the left hemisphere [14]. In contrast, the valence hypothesis posits that the right hemisphere is primarily involved in processing negative emotional stimuli (e.g., fear or sadness), whereas the left hemisphere is more engaged in the processing of positive emotional stimuli [13]. While previous research employing functional magnetic resonance imaging or event‐related potentials (ERP) has provided support for both hypotheses [15–17], the differential responses of the left and right M1 to negative emotional scene stimuli have not been reported. A recent study using TMS found that the excitability of the right M1 was significantly greater than that of the left M1 during the early recognition of emotional faces [8]. However, the involvement of the right M1 in more complex scene‐based emotional processing remains unclear. Compared with emotional face stimuli, emotional scene stimuli convey richer semantic content and greater survival relevance, which may elicit distinct lateralization patterns in M1 excitability.

Single‐pulse TMS is a non‐invasive technique used to assess motor cortex excitability by measuring MEPs recorded from contralateral hand muscles [18]. When TMS is applied at different time points prior to action execution, it enables the tracking of corticospinal excitability during the motor preparation phase without being affected by actual motor output [19, 20]. Owing to its high temporal resolution, TMS has been widely employed to investigate the role of the M1 in threat detection, emotional evaluation, and action tendencies. Previous studies have demonstrated that when single‐pulse TMS is delivered to M1 within 100–300 ms following the onset of emotional stimuli, changes in MEP amplitude can be observed in the target muscles [12, 21]. These findings support the utility of TMS as an effective method for probing the involvement of M1 in the temporal dynamics of emotional processing.

Therefore, this study examined the excitability of the left and right M1 at early, intermediate, and late time windows following the presentation of emotional scene stimuli with different valences. The objective was to characterize the dynamic modulation of motor cortex excitability induced by negative emotional scene stimuli and to investigate the distinct functional roles of the left and right M1. Emotional stimuli with negative, neutral, and positive valences were used as experimental materials. Single‐pulse TMS was applied to either the left or right M1 at 150, 250, and 350 ms after stimulus onset. Corticospinal excitability was assessed by recording MEPs from the hand muscles. We hypothesized that, in the early time window (150 ms post‐stimulus), negative emotional scene stimuli would elicit greater MEP amplitudes in the right M1 compared to positive emotional stimuli. In the late time window, the extent of MEP enhancement induced by negative and positive stimuli was expected to be comparable.

Finally, socially emotional stimuli may also elicit negative coping strategies, such as threat avoidance. Previous research has indicated that motor responses during social perception are modulated by an individual’s predisposition toward negative coping [6]. Accordingly, the present study further investigated whether individual differences in negative coping tendencies could predict the magnitude of motor responses to emotional scene stimuli.

## 2. Methods

### 2.1. Participants

Twenty‐eight undergraduate students (14 women; mean age ± standard deviation: 21.79 ± 2.10 years) were recruited from Shanghai University of Sport. All participants were right‐handed (assessed via a standard handedness inventory [22]) and had no TMS contraindications. Participants had normal or corrected‐to‐normal visual acuity. None had a history of psychiatric or neurological disorders. 1 day prior to the experiment, participants were instructed to avoid consuming caffeinated beverages and to refrain from staying up late to minimize potential factors that might influence motor cortex excitability.

The procedure was approved by the Ethics Committee of Shanghai University of Sport. Participants provided informed consent prior to the experiment, and none reported any discomfort or adverse effects during the TMS sessions.

### 2.2. Visual Stimuli

Ninety pictures were selected from the Chinese Affective Picture System (CAPS) [23], including 30 negative, 30 positive, and 30 neutral scenes (Table 1), with each category matched for visual complexity and brightness. Example CAPS pictures are presented in Figure 1A. A one‐way analysis of variance (ANOVA) conducted on the three picture categories (positive, negative, and neutral) revealed significant differences in their rated valence (*F*_(2,123)_ = 49.50, *p* < 0.001) and arousal levels (*F*_(2,123)_ = 8.95, *p* < 0.001). Post hoc analyses confirmed that there were significant differences in the emotional valence between each of any two picture types (for both comparisons, *p* < 0.001). Furthermore, arousal levels of positive and negative pictures were both significantly higher than those of neutral pictures (both *p* < 0.001).

Figure 1Procedure for the emotion recognition task. (A) Examples of visual stimuli. (B) Trial screen sequence overview. TMS, transcranial magnetic stimulation.

**Figure:**
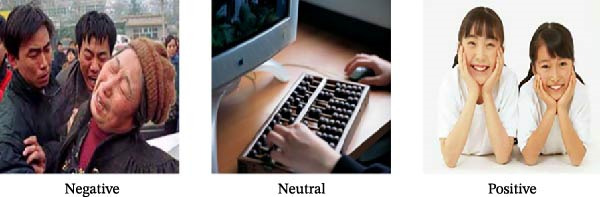
(A)

**Figure:**
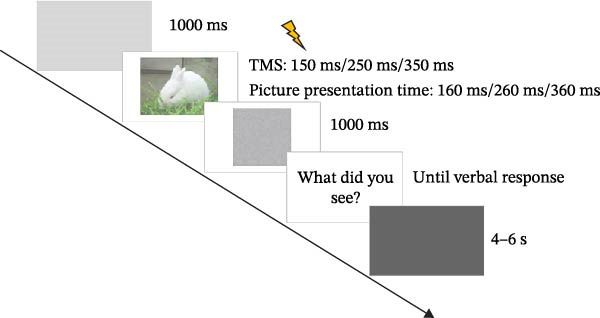
(B)

**Table 1:** Valence and arousal of emotional pictures.

Type	Valence	Arousal
Negative pictures	3.10 ± 0.27	5.33 ± 0.27
Neutral pictures	4.88 ± 0.12	4.10 ± 0.24
Positive pictures	6.10 ± 0.23	5.36 ± 0.21

### 2.3. Experimental Tasks

Presentation of CAPS images and TMS triggering were controlled using MATLAB software (R2018b). Participants were seated ~50 cm from the computer screen. Each trial proceeded through the following sequence: (1) The trial commenced with a gray background displayed for 1000 ms. (2) A randomly selected emotional picture was then presented. Each image was displayed in a random order for a randomly selected duration of 160, 260, or 360 ms. (3) Following the disappearance of the emotional picture, a mosaic of random dots was displayed for 1000 ms (as a visual mask intended to reduce the influence of afterimages on task accuracy). Subsequently, a prompt appeared on the screen reading, “What picture did you see?” Participants then verbally classified the emotional picture they had observed. A researcher recorded these verbal responses using a keyboard. Negative pictures were coded as 1, neutral images as 2, and positive images as 3. Participants were instructed to respond verbally and promptly to the on‐screen question. This guided verbal response paradigm was adopted to minimize excitatory confounds from prolonged language processing. (4) After each response, an inter‐trial interval consisting of a black screen was displayed for 4–6 s before the next trial began. This active classification of emotional images, rather than passive viewing, was employed because prior research suggests it increases the likelihood of detecting specific emotional regulation effects in various brain regions, including the motor system, as observed in previous imagination and ERP studies. To minimize potential fatigue effects on the results, the emotion classification task was divided into two blocks, with participants afforded a sufficient rest period between blocks. Each block comprised 45 trials.

### 2.4. TMS

A figure‐eight‐shaped stimulation coil connected to a Magstim 200 stimulator (Magstim, Whitland, Dyfed, UK) was placed over the right or left M1. The coil was positioned tangentially to the scalp, with the handle facing backward and forming a 45° angle with the mid‐sagittal line. The coil position was adjusted to locate the motor hotspot, defined as the site consistently yielding the largest MEP in the contralateral first dorsal interosseous (FDI) muscle. This M1 motor hotspot was marked to ensure consistent coil placement throughout the experiment. The magnetic pulse intensity was set at 120% of the resting motor threshold. Resting motor threshold was defined as the minimum stimulator output intensity required to produce MEPs with an amplitude of at least 50 μV in the contralateral FDI muscle with 50% probability.

The experimental procedure was divided into two phases: a baseline testing phase and a task testing phase. During the baseline testing phase, MEPs were collected using single‐pulse TMS to assess baseline motor cortex excitability. For this baseline measurement, participants were instructed to close their eyes slightly and imagine watching a sunset on a beach, maintaining a relaxed, natural state. Hand muscles were kept relaxed, and single TMS pulses were delivered every 10 s.

During the task phase, each picture was presented for a randomly selected duration of 160, 260, or 360 ms. A single TMS pulse was delivered to either the left or right M1 at 150, 250, or 350 ms after picture onset. These TMS timings corresponded to the final 10 ms of the 160, 260, and 360 ms picture presentation durations, respectively. This procedure ensured that M1 excitability was assessed while visual information was being processed. The inter‐trial interval between TMS pulses was greater than 10 s to minimize potential cumulative effects of TMS on motor cortex excitability.

Participants completed two experimental sessions to enable electromyography (EMG) recordings from both the left and right FDI muscles. In one session, TMS was applied to the left M1 with EMG recorded from the right FDI, and in the other session, TMS was applied to the right M1 with EMG recorded from the left FDI. The order of these sessions (left M1 stimulation first vs., right M1 stimulation first) was counterbalanced across participants. The interval between the two sessions was 20 min.

### 2.5. EMG

Surface EMG signals were recorded using 9‐mm‐diameter Ag–AgCl surface electrodes. The active electrode was placed over the belly of the FDI muscle, parallel to the direction of muscle fibers. The reference electrode was positioned ~3 cm distally, over the tendon. A ground electrode was placed on the wrist. The EMG signal was amplified (×1000), bandpass filtered (20 Hz–2.5 kHz), digitized at 5 kHz using an analog‐to‐digital interface (Micro1401; Cambridge Electronics Design, Cambridge, UK), and stored on a computer for offline analysis.

### 2.6. Data Analysis

Accuracy in the classification task was analyzed using a two‐way repeated‐measures ANOVA, with hemisphere (two levels: left or right) and TMS onset time (three levels: 150, 250, or 350 ms) as within‐subjects factors. Post hoc comparisons were performed using Bonferroni corrections.

Mean MEP amplitudes for each condition were expressed as ratios of the mean peak‐to‐peak MEP amplitude at baseline (condition/baseline). Because background EMG can modulate MEP amplitudes, pre‐TMS EMG was assessed by calculating the mean rectified signal across a 100‐ms interval prior to TMS. MEPs with preceding background EMG deviating from the mean by more than three standard deviations were removed from further analysis. To examine how neural responses to emotional scenes build up gradually, a three‐way repeated‐measures ANOVA was conducted for MEP ratios with hemisphere (two levels: left or right), picture valence (three levels: negative, neutral, or positive), and TMS onset time (three levels: 150, 250, or 350 ms) as within‐subjects factors. The Greenhouse‐Geisser method was used to correct for violations of sphericity. Paired *t*‐tests with Bonferroni corrections for multiple comparisons were used for post hoc analysis if ANOVAs showed significant interactions among factors. *p* < 0.05 was considered significant. SPSS version 22 (IBM Corp., Armonk, NY) was used for statistical analyses.

To investigate the relationship between motor reactivity and coping style, partial correlation analyses were performed between specific MEP measures (derived from recordings at different TMS onset times) and scores on the two subscales of the Simplified Coping Style Questionnaire.

## 3. Results

### 3.1. Classification Accuracy of CAPS Pictures

**Figure 2:**
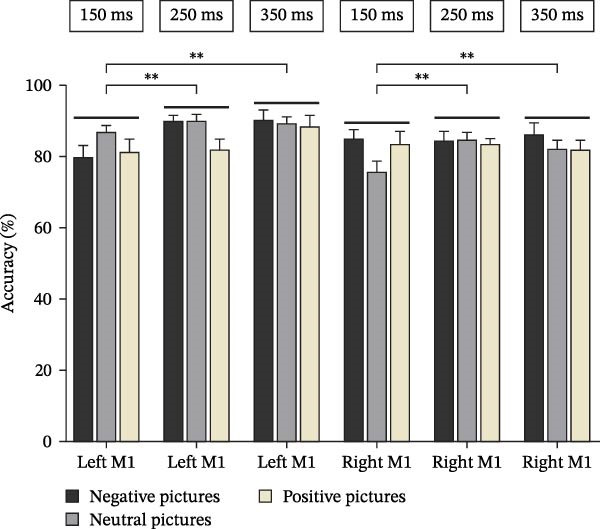
Accuracy across the three time points and hemisphere.  ^∗∗^ *p* < 0.01 for significant differences between time points within each hemisphere. M1, primary motor cortex.

Accuracy data for the emotional picture classification task were calculated (mean accuracy for each emotional picture type is presented in Figure 2). Normality testing of these overall accuracy data indicated a non‐normal distribution (*p* < 0.05). Therefore, the accuracy data were logarithmically transformed prior to statistical analysis. The transformed accuracy data were then analyzed using a two‐way repeated‐measures ANOVA with hemisphere (left, right) and TMS onset time (150, 250, 350 ms) as within‐subjects factors.

The ANOVA revealed no significant main effect of hemisphere (*F*_(1, 83)_ = 2.78, *p* =  0.099) and no significant hemisphere × TMS onset time interaction (*F*_(2, 166)_ = 2.04, *p* =  0.138). However, there was a significant main effect of TMS onset time (*F*_(2, 166)_ = 8.33, *p* =  0.001). Post hoc comparisons (Bonferroni corrected) indicated that classification accuracy when TMS was delivered at 150 ms was significantly lower than when TMS was delivered at 250 ms (*p* =  0.01) and at 350 ms (*p* =  0.001).

### 3.2. Neurophysiology

A paired‐sample *t*‐test indicated no significant difference in baseline MEP amplitudes (*t*_(27)_ = 1.04, *p* =  0.307) between the sessions involving right M1 and left M1 stimulation.

**Figure 3:**
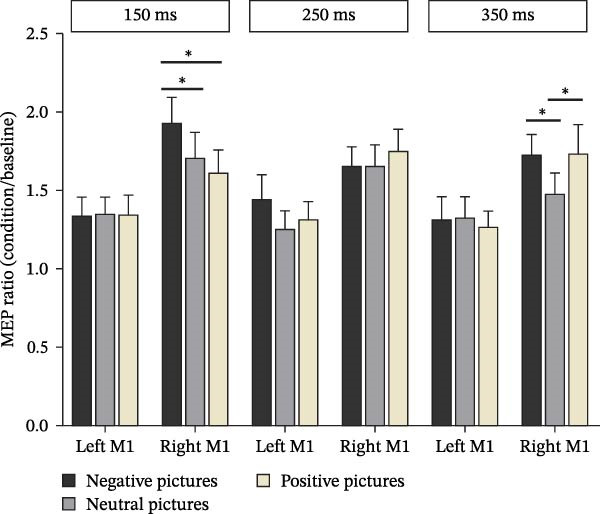
MEP ratios recorded at 150, 250, and 350 ms following the presentation of negative, neutral, and positive pictures during TMS sessions targeting the left M1 and right M1, respectively.  ^∗^ *p* < 0.05 for significant differences between different valence categories (negative vs., neutral vs., positive) in the right M1 at 150 and 350 ms. M1, primary motor cortex. MEP ratios represent normalized corticospinal excitability relative to baseline. Asterisks indicate statistically significant differences between valence conditions following Bonferroni correction.

To specifically investigate the effects of TMS onset time and visual conditions on motor excitability, MEP ratios were analyzed using a repeated‐measures ANOVA with hemisphere (left M1 stimulation, right M1 stimulation) × TMS onset time (150, 250, or 350 ms) × picture valence (negative, neutral, or positive) as within‐subject factors (Figure 3). The ANOVA revealed a significant main effect of hemisphere (*F*_(1,27)_ = 6.94, *p* =  0.014), with MEP ratios following right M1 stimulation being significantly higher than those following left M1 stimulation. There was also a significant main effect of picture valence (*F*_(2, 54)_ = 7.47, *p* =  0.001). Post hoc comparisons (Bonferroni corrected) indicated that MEP ratios elicited during the observation of negative pictures were significantly higher than those for neutral pictures.

A significant three‐way interaction was found among hemisphere × TMS onset time × picture valence (*F*_(4, 108)_ = 4.52, *p* =  0.002). Post hoc analysis of this interaction revealed specific effects for right M1 stimulation. Specifically, at the 150 ms TMS onset time, MEP ratios following right M1 stimulation were significantly higher for negative pictures compared to both positive pictures (*p* =  0.001) and neutral pictures (*p* =  0.036). Furthermore, at the 350 ms TMS onset time, MEP ratios following right M1 stimulation were significantly greater for negative pictures (*p* =  0.016) and positive pictures (*p* =  0.039) compared to neutral pictures. No significant differences in MEP ratios related to picture valence were found for right M1 stimulation at the 250 ms TMS onset time.

### 3.3. Relation Between Personality and Motor Reactivity

**Figure 4:**
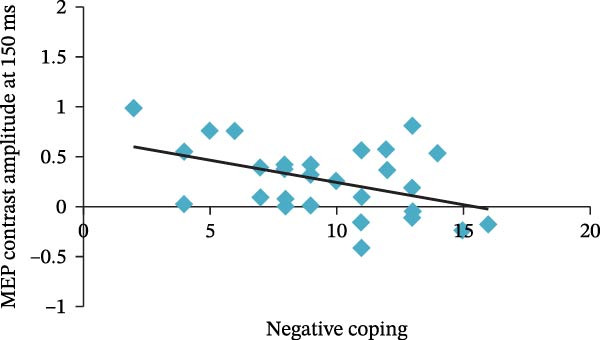
Correlation between MEP contrast amplitude at 150 ms and negative coping subscale score of the Simplified Coping Style Questionnaire.

An index of early motor reactivity for negative pictures (MEP contrast computed at 150 ms) was entered into a partial correlation analysis with the two subscales of the Simplified Coping Style Questionnaire (Figure 4). The partial correlation analysis revealed that the Negative Coping subscale was significantly related to this MEP contrast (*r* = −0.375, *p* =  0.024).

## 4. Discussion

This study investigated the response of the motor system to emotional scenes. The present findings demonstrate that motor system responses to emotional stimuli are predominantly lateralized to the right hemisphere. Specifically, when participants viewed emotional pictures, corticospinal excitability in the right M1 was significantly greater than that in the left M1. Moreover, the temporal dynamics of motor system responses varied across different TMS onset windows. In an early time window (150 ms after picture onset), negative emotional pictures elicited greater excitability in the right M1 compared to positive and neutral pictures. In the later time window (350 ms after picture onset), both positive and negative emotional pictures elicited greater excitability in the right M1 compared to neutral pictures. These findings provide insight into the neural basis of emotion‐related modulation of the motor system and may offer a potential avenue for future research into alterations of these processes in mood disorders such as depression and anxiety. For clarity, in the present study, “motor reactivity” refers to changes in corticospinal excitability indexed by MEP amplitude, “motor facilitation” denotes relative increases in this excitability compared to baseline, and “motor bias” is used more broadly to describe valence‐related asymmetries in these responses.

The right hemisphere hypothesis posits that the right hemisphere is predominantly involved in emotional processing relative to the left [14, 24, 25]. Supporting this view, studies combining low‐resolution brain electromagnetic tomography and ERPs have shown that emotional states are associated with dynamic patterns of brain activation, with peak activity observed in the right frontal cortex during critical time windows [26]. This finding underscores the role of the right hemisphere in emotional cognition. In addition, recent evidence has demonstrated functional asymmetries in the ventromedial prefrontal cortex, further supporting right‐lateralized emotional processing [27, 28]. At the motor level, Giovannelli et al. [29] reported that emotional stimuli can induce greater excitability in the right M1. The present findings are consistent with this right‐hemisphere dominance at the level of the motor cortex, as evidenced by significantly higher MEP ratios following right M1 stimulation compared to left M1 stimulation. Importantly, these results suggest that right‐hemisphere dominance extends beyond perceptual and evaluative processes to include modulation of motor system excitability in response to emotional stimuli.

When examining the recognition accuracy in the experimental results, it was found that applying TMS at 150 ms significantly reduced the participants’ recognition accuracy for all images compared to TMS applied at 250 and 350 ms. This finding is consistent with prior studies [9, 30]. Previous research has demonstrated that TMS applied to the right M1 at 150 ms following stimulus onset selectively disrupts visual recognition of body expressions, resulting in significantly lower recognition accuracy at the early stage (150 ms) than at the later stage (300 ms). Researchers have suggested that the right M1 may reflect the spillover of somatosensory activity associated with emotional perception, and that TMS interference propagates to the closely connected right somatosensory cortex, thereby impairing emotional perception and recognition [9, 30]. The present study extends this finding by broadening the research scope of TMS interference from body expression recognition to the recognition of emotional scenes. It provides a new perspective for investigating the interaction mechanisms between the motor cortex and somatosensory regions in the processing of emotional information.

Perception and immediate response to potential threats are critical for individual survival [4]. Negative stimuli must more strongly and urgently recruit perceptual processing and motor resources in order to minimize the adverse consequences associated with aversive cues. As a result, individuals exhibit a “negative bias” in the processing of emotional information. Behavioral studies have linked negative motor bias to antagonistic responses elicited by threatening stimuli, while at the neural level, such effects are associated with stronger and earlier modulation of corticospinal excitability, reflecting prioritized processing of threat‐related information [1, 2, 31]. A similar phenomenon has been observed at the electrophysiological level. Recent studies employing intracranial ERP techniques to examine amygdala responses to negative stimuli in patients with epilepsy have demonstrated that unpleasant stimuli are detected more rapidly (latency 100–200 ms) compared with pleasant or neutral stimuli [32, 33]. This “negative bias” has also been explored at the level of the motor cortex and has been substantiated by numerous TMS studies [8, 9, 12]. Borgomaneri et al. applied monopulse TMS to the M1 and found that at 150 ms following the presentation of emotional stimuli, motor cortex excitability increased when participants viewed negative natural scenes, in comparison to positive or neutral scenes. This suggests that negative emotional events more urgently recruit motor system activity, such that viewing negative emotional stimuli during rest automatically triggers short‐latency activation of the dominant‐hand motor representation [12]. In our study, we observed that when TMS was applied at 150 ms, the MEP ratio in the right M1 was significantly higher for negative images than for neutral and positive images. Both behavioral and electrophysiological evidence indicate that negative stimuli are more likely to induce rapid motor system responses in observers [26, 34, 35]. These findings support the evolutionary perspective that rapid neural responses to threats contribute to the survival of the organism.

In addition to threat‐related prioritization, alternative mechanisms underlying the early modulation of right M1 excitability warrant further consideration. One plausible account involves rapid feedforward visuomotor coupling, whereby coarse visual information is transmitted via fast‐processing pathways to motor‐related regions, enabling early‐stage integration of perceptual and motor signals [36, 37]. Such coupling may be supported by subcortical routes (e.g., the superior colliculus–pulvinar pathway), which facilitate the rapid detection of biologically salient stimuli prior to full cortical appraisal [38, 39]. Furthermore, the observed early effects may also be influenced by low‐level perceptual salience inherent in negative scenes, including features such as contrast, motion‐related cues, or threat‐relevant configurations [40]. These properties may preferentially capture attentional resources and enhance early sensory gain, which may in turn propagate to motor cortical areas. From a broader perspective, this early modulation may reflect a rapid and distributed integration process across perceptual, affective, and motor systems [41, 42], rather than a strictly motor‐specific response. Within predictive processing frameworks, such early changes in corticospinal excitability may index the updating of action‐relevant predictions in response to salient environmental cues [43], even in the absence of overt motor output.

In the late time window (350 ms after picture onset), both positive and negative pictures elicited significantly greater motor facilitation in the right M1 compared to neutral pictures. This finding suggests that emotional information from natural scenes can dynamically regulate the functional state of the human corticospinal system. Previous research has indicated that for MEPs collected at 300 ms or later post‐stimulus onset—across various tasks such as passive observation, active classification, or task‐irrelevant viewing of images—positive and negative images often exert a similar facilitatory effect on the motor cortex. For instance, Borgomaneri et al. reported comparable findings during passive presentation of emotional visual scenes and emotionally congruent auditory stimuli [9, 21]. Research using ERPs has focused on exploring the temporal dynamics of emotion processing, and these studies have found that electrophysiological responses to positive and negative images can be similar in the 300–600 ms range [10]. The present study employed TMS to directly investigate the dynamic response of the motor system to emotional stimuli, and our results for the late time window are consistent with these previous findings. A possible explanation is that in this later processing stage, as individuals evaluate emotional pictures of differing valences [6], emotional information is integrated at a higher level, resulting in modulation of the motor system that reflects evaluative processing rather than direct preparation for action. Consequently, this evaluative and preparatory processing for both positive and negative emotional pictures may lead to an enhancement of M1 excitability.

No significant modulation of motor system excitability in response to emotional images was observed during the mid‐latency time window (250 ms). One possible explanation is that this time point corresponds to the N2 component window. Studies on emotion‐related ERPs have shown that both positively valenced and neutral stimuli elicit higher N2 amplitudes, supporting the notion that these stimuli do not require the urgent allocation of attentional resources to the same extent as negative stimuli [34]. In the present study, no modulation of motor cortex excitability by emotional stimuli was found at this latency, which may be attributable to differences in experimental paradigms. The N2 component is typically associated with the allocation of attention to novel and potentially salient stimuli. Therefore, previous research has often employed the oddball paradigm or its variants. Under such paradigms, when deviant stimuli are presented, the N2 component exhibits the highest amplitude, typically peaking around 250 ms [34, 35]. In the current study, an emotional evaluation task was employed, and emotional images were not treated as novel or deviant stimuli. As a result, the motor cortex did not exhibit a measurable response to emotional stimuli during this stage. Importantly, the absence of significant modulation at this latency should not be interpreted as evidence for a lack of motor system involvement, but may instead reflect task‐specific or temporal sensitivity limitations of the current paradigm.

The present study found that early motor reactivity to negative pictures, defined as the MEP contrast computed at 150 ms, was associated with scores on the Negative Coping subscale of the Simplified Coping Style Questionnaire. This finding complements existing theories of emotion–motor interaction. While it is well established that emotional stimuli can modulate motor system excitability, the current results further suggest that individual differences in coping style, as a personality‐related trait, may influence early motor system responses to negative emotional stimuli [6, 30]. Specifically, a tendency towards negative coping, viewed as a habitual response pattern, appears to modulate motor system activity during the initial stages of emotional perception, thereby offering a new perspective on the mechanisms underlying emotion‐driven motor processes. Importantly, these effects are likely to emerge from dynamic interactions across perceptual, affective, and motor systems, rather than from a single localized mechanism, highlighting the distributed and context‐dependent nature of emotion–motor integration [44–46]. Furthermore, this finding highlights the importance of individual differences in emotional perception and motor system responses, suggesting that individuals may exhibit distinct patterns of motor cortex modulation when exposed to the same negative stimuli, depending on their coping tendencies. Future research could therefore explore individual variability in motor system responses across different emotional contexts in conjunction with a broader range of personality traits [30]. Such work would contribute to a more comprehensive understanding of the complexity of the emotion–motor system and may provide a theoretical basis for the development of personalized psychological interventions. For instance, these insights could guide the design of tailored intervention programs for individuals with a strong tendency towards negative coping. However, this finding should be interpreted as exploratory given the relatively small sample size, and it does not support strong conclusions regarding stable trait‐level neural markers. Future studies with larger samples are required to validate this association.

The current study used healthy participants, and future research could explore whether individuals with depression or anxiety show altered right M1 excitability during negative scene processing, which may provide a potential direction for future research exploring neurophysiological alterations in clinical populations such as depression and anxiety.

It should be noted that MEP amplitude reflects changes in corticospinal excitability within the motor system and does not directly index overt motor behavior. Therefore, the present findings should be interpreted as neurophysiological correlates of emotional processing at the motor system level, rather than evidence of actual motor preparation or action tendencies.

Several limitations should be acknowledged. First, the sample consisted of young, healthy, and right‐handed individuals, which may limit the generalizability of the findings. Second, the use of TMS‐derived MEPs provides an indirect index of motor system involvement and does not capture overt behavioral responses. Third, the relatively small sample size, particularly for individual differences analyses, warrants cautious interpretation.

## 5. Conclusion

The motor system’s response to emotional stimulation was primarily observed in the right hemisphere. In the early time window, negative emotional stimuli elicited a stronger response in the right M1, whereas in the later time window, both positive and negative emotional stimuli elicited comparable responses in the right M1.

## Author Contributions

**Haijiang Meng:** conceptualization, methodology, writing original draft, and writing, review and editing. **Xiaoxiao Zhang:** data curation, visualization, investigation, and writing, review and editing. **Jian Zhang**: conceptualization, methodology, and writing, review and editing. **Chaoying Zheng**: project administration, supervision, writing original draft, and writing, review and editing.

## Funding

The present study was funded by Social Science Research Projects of Shaoxing University in China (Grant No. 13011002002/176).

## Disclosure

The funding source had no role in the study design, data analysis and interpretation, report preparation, or publication decision‐making.

## Conflicts of Interest

The authors declare no conflicts of interest.

## Data Availability

The supporting data for this study are available from the corresponding authors upon reasonable request.
